# Regulation of mesenchymal stem cell differentiation by autophagy

**DOI:** 10.1515/med-2024-0968

**Published:** 2024-05-21

**Authors:** Yanan Wei, Zejun Zheng, Ying Zhang, Jinmeng Sun, Shuangshuang Xu, Xinsheng Di, Xiaoling Ding, Gang Ding

**Affiliations:** School of Stomatology, Shandong Second Medical University, Weifang, 261053, Shandong, China; Clinical Competency Training Center, Shandong Second Medical University, Weifang, 261053, Shandong, China

**Keywords:** autophagy, differentiation, mesenchymal stem cells, regenerative medicine

## Abstract

Autophagy, a process that isolates intracellular components and fuses them with lysosomes for degradation, plays an important cytoprotective role by eliminating harmful intracellular substances and maintaining cellular homeostasis. Mesenchymal stem cells (MSCs) are multipotent progenitor cells with the capacity for self-renewal that can give rise to a subset of tissues and therefore have potential in regenerative medicine. However, a variety of variables influence the biological activity of MSCs following their proliferation and transplantation *in vitro*. The regulation of autophagy in MSCs represents a possible mechanism that influences MSC differentiation properties under the right microenvironment, affecting their regenerative and therapeutic potential. However, a deeper understanding of exactly how autophagy is mobilized to function as well as clarifying the mechanisms by which autophagy promotes MSCs differentiation is still needed. Here, we review the current literature on the complex link between MSCs differentiation and autophagy induced by various extracellular or intracellular stimuli and the molecular targets that influence MSCs lineage determination, which may highlight the potential regulation of autophagy on MSCs’ therapeutic capacity, and provide a broader perspective on the clinical application of MSCs in the treatment of a wide range of diseases.

## 1 Introduction

The repair of damage to cells and organs caused by a wide range of diseases and toxic factors has been a major challenge in medicine. Increasing evidence suggested that mesenchymal stem cells (MSCs), which have demonstrated excellent potential in a wide range of disease therapies and injury repair [1], offer tremendous advantages in addressing many of the medical problems faced by humanity. Pre-treatment with a variety of endogenous and exogenous factors can repair the impaired biological functions of MSCs, which can help improve their survival and proliferation rates and provide better therapeutic outcomes for clinical treatment.

Being found in most tissues or organs, adult stem cells, which have the potential to differentiate into pluripotent cells, play an essential role in the normal renewal and maintenance of the human body. These adult stem cells ensure the normal maintenance of tissues by rapidly replacing degenerating stem cells. This degeneration–regeneration cycle rejuvenates the tissues and helps maintain tissue function [2]. Bone marrow-derived MSCs (BMSCs) are widely used in a variety of research applications due to their strong capacity for progeny expansion, multidirectional differentiation, haematopoietic support, and immunomodulation [3], and are the focus of this article’s description of MSCs. This versatile and modifiable differentiation potential of MSCs, as well as their ability to secrete a variety of trophic factors and modulate the immune system of recipients, make them a promising source of cells for regenerative medicine [4].

Recent studies have revealed that autophagy has a fundamental biological role in the maintenance of the regenerative capacity of MSCs and bone homeostasis. Autophagy is associated with the modulation of MSCs differentiation, whereas autophagy dysfunction impairs MSCs function, leading to imbalanced bone remodelling as well as extensive senescence [5]. There is a complex association between autophagy, induced by various extracellular or intracellular signals, and molecular targets that affect MSCs differentiation and self-renewal [6]. In human MSCs, a consistent assay of LC3-I to LC3-II transformation rates suggests that activation of autophagic fluxes and commitment of MSCs to a variety of cell lines is dependent on basal autophagic activity [7]. There is evidence of reduced accumulation of undegraded autophagosomes and autophagic turnover in undifferentiated MSCs, in contrast to stimulation of osteoblast differentiation, which leads to a steady increase in turnover rates [4].

In order to better elucidate how autophagy affects the differentiation therapeutic properties of MSCs, we outlined research progress on the potential connection between the differentiation characteristics of MSCs and their regulation of autophagy, and summarise cases of diseases in which differentiation is associated with such regulation, thereby providing a wider vision for the medical application of MSCs in the treatment of various diseases.

## 2 Autophagy

Autophagy involves delivering cytoplasmic cargo to the lysosome to be degraded and can be classified into at least three categories depending on how the cargo is delivered to the lysosome: chaperone-mediated autophagy, micro-autophagy, and macroautophagy [8]. Despite the differences in their morphologies, all three types of autophagy ultimately involve the degradation and recycling of cargo within the lysosome. Chaperone-mediated autophagy involves the binding of cargo proteins with specific pentapeptide motifs to molecular chaperones, which are then translocated into the lysosomal lumen. Micro-autophagy is the process by which the lysosome membrane is directly invaginated to encapsulate and degrade the cargo. Macroautophagy separates cargo through double membrane-bound vesicles. These vesicles subsequently fuse with the lysosome and destroy its contents. The lysosomal degradation pathway of autophagy, is essential for preserving intracellular homeostasis. This includes preventing genomic damage, responding to metabolic stress, and eliminating dangerous cargoes like misfolded proteins, broken organelles, and intracellular pathogens [9].

**Figure 1:**
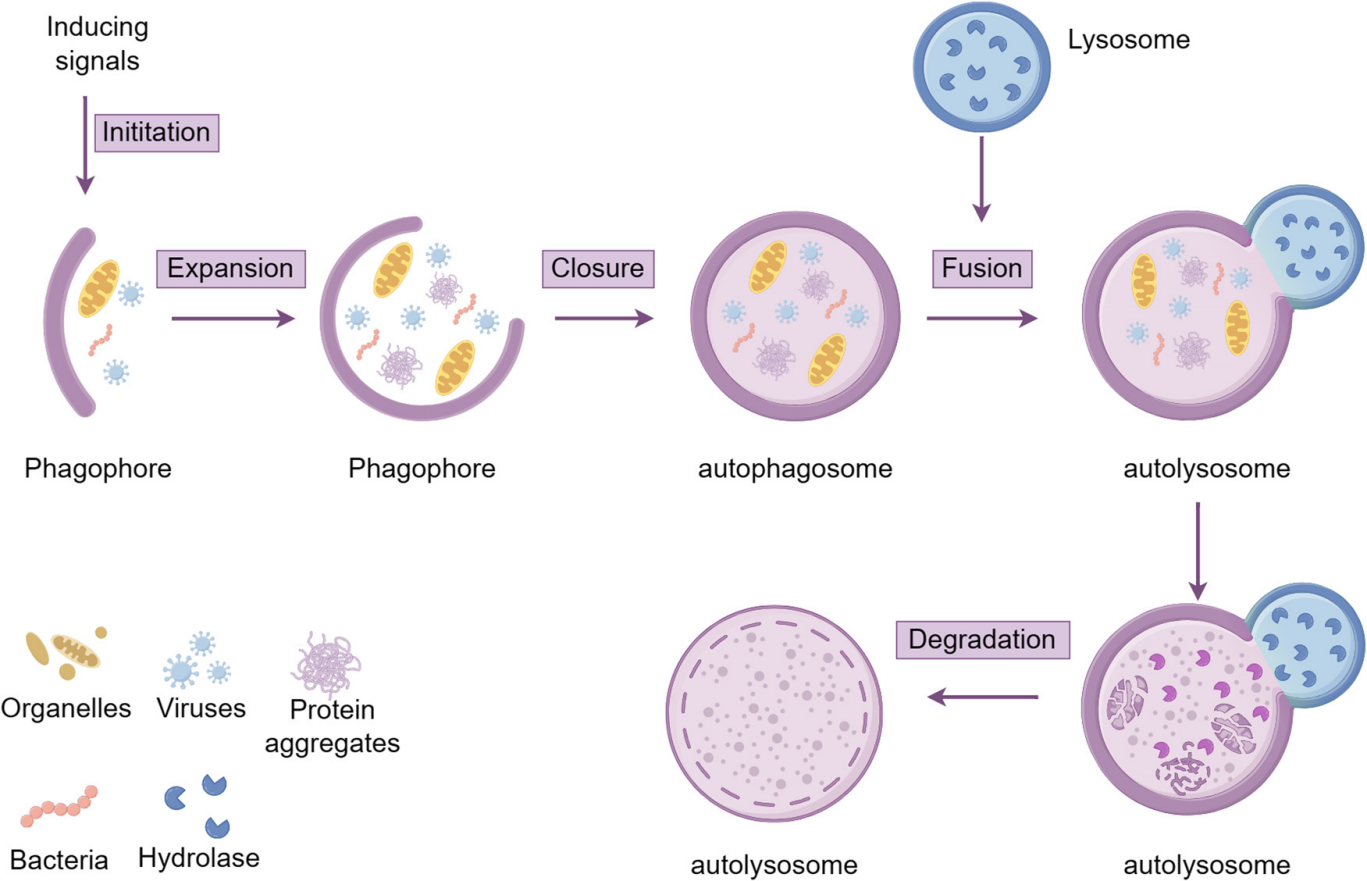
Schematic for autophagy. The process of autophagy development involves the following main steps: initiation, expansion, formation of autophagosomes, fusion of autophagosomes with lysosomes, and degradation of autolysosome contents. This figure was generated by Figdraw.

In the typical form of autophagy, the process of autophagy begins with the emergence of a phagocytic membrane. After receiving an autophagy-inducing signal, the cell forms a small, flattened, double-membrane structure somewhere in the cytoplasm that can be observed by electron microscopy, called a phagophore [10], which continually swells and extends to take in a number of intracytoplasmic components, such as organelles, proteins, and bacterial viruses, and eventually closes to form a double-membrane autophagosome. Although the origin of the membrane is unknown, a number of studies have proposed Golgi complex, mitochondria, endoplasmic reticulum, and plasma membrane as potential candidates [11]. The lysosome and the formed autophagosome fuse at this point, causing the autophagosome’s inner membrane to be broken down by lysosomal enzymes as well as the autophagosome’s contents to enter the lysosome and be broken down, allowing the cell to recycle materials into the cytosol [12] (Figure 1).

### 2.1 Autophagy mechanism

**Figure 2:**
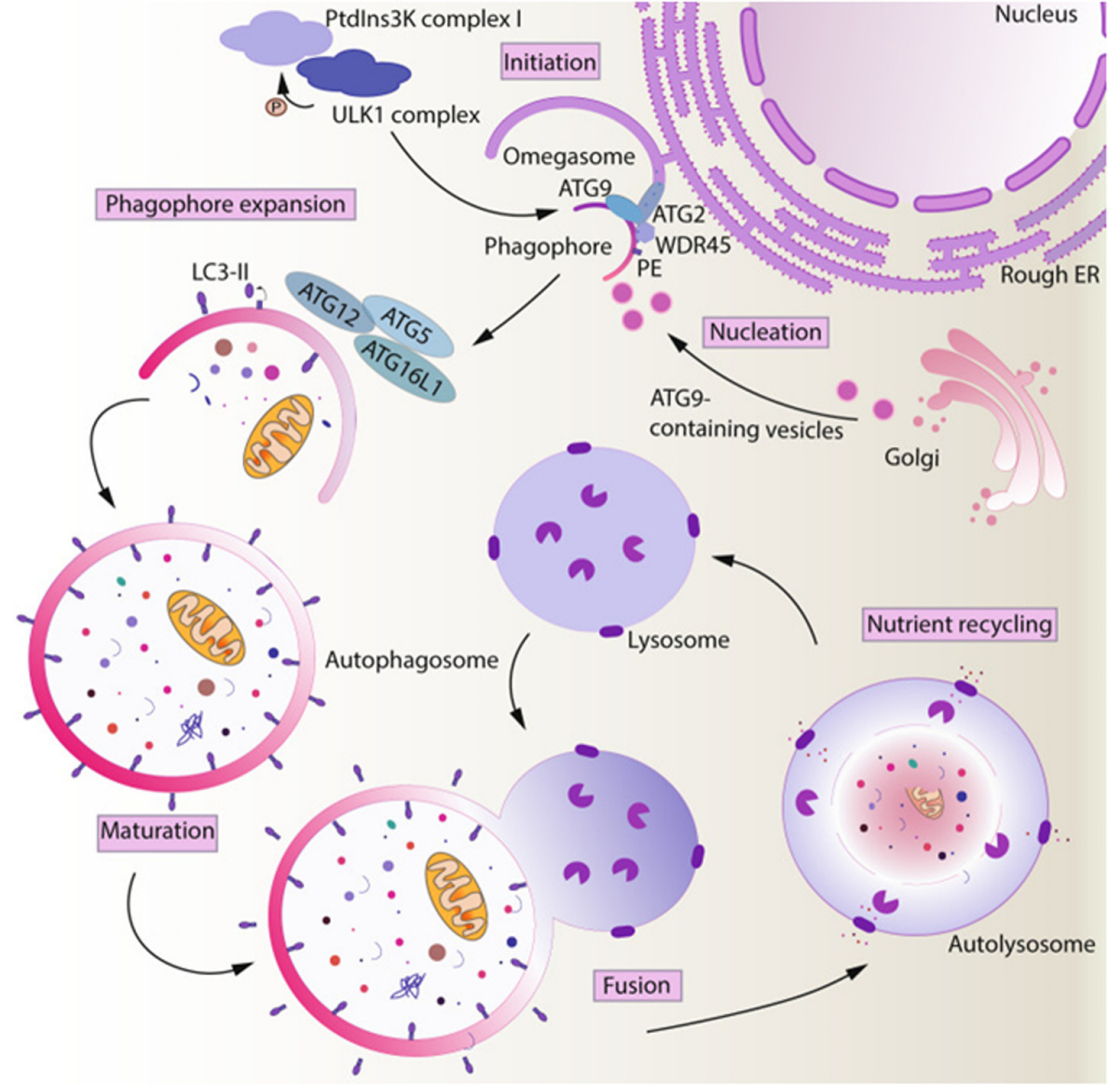
Process of autophagy begins with the induction of ULK1 and PIK3C3/Vps34 complexes, which initiate nucleation of the omegasome. The membrane constituting the phagophore is delivered by vesicles containing Atg9. Amplification of the autophagic membrane involves a coupling system of two ubiquitin-like proteins, and finally, the autophagosome fuses with the lysosome to form an autolysosome, which allows degradation of its contents. Reprinted from ref. [12], Copyright (2021), Cellular & Molecular Immunology.

Autophagy is regulated by more than 30 highly conserved autophagy-related genes (Atg), which were originally identified in a yeast genetic screen [13]. One of the Atg core proteins, Atg8, is considered to be a marker of membrane dynamics during autophagy due to its simultaneous location at both the separating membrane and the autophagosome [14]. A point dot close to Atg8 is known to be a phagocytic assembly site, which can initiate autophagy by aggregating Atg proteins and assembling them. In mammals, autophagy is initiated primarily through two complexes: the ULK1 complex (unc-51-like autophagy-activated kinase) and the class III PI3 kinase complex I (III phosphatidylinositol 3-kinase). As a serine–threonine kinase complex that includes ULK1/2, FIP200 (focal adhesion kinase family interacting protein of 200 kD), Atg 13, and Atg 101, ULK1 complex induces the expression of Beclin-1, which leads to the formation of a complex of Beclin-2, VPS34 (Vacuolar Protein Sorting 34), VPS15 (Vacuolar Protein Sorting 15), and Atg14L to form the class PI3K VPS34 complex. Two different Beclin class 1/III phosphatidylinositol 3-kinase (PI3KC3) complexes produce phosphatidylinositol 3-phosphate to participate in autophagosome nucleation (PI3KC3-C1 involves Beclin 1, VPS34, VPS15, and Atg14) or in endolysosomal and autophagic lysosome maturation (PI3KC3-C2 involves Beclin 1, VPS34, VPS15, and UVRAG). Vesicles containing Atg9A (the only transmembrane core Atg protein) provide membranes for autophagosomes. The source for the formation of autophagosomes can be from the endoplasmic reticulum, the Golgi complex, mitochondria, or through plasma membrane-mediated endocytosis. The amplification of autophagic membranes involves two coupling systems of ubiquitin-like proteins, the first of which involves the formation of the Atg12–Atg5–Atg16 complex. The coupling of Atg12 to Atg5 is dependent on a common E1-like enzyme, Atg7, and a specific E2-like enzyme, Atg10, and culminates in the coupling of the c-terminal glycine of Atg12 to the side chain of the Lys149 of Atg5 [15], the uncoupling enzyme is not present in the Atg12–Atg5 system and the formation of this coupling is constitutive. The Atg12–Atg5 coupling further interacts with Atg16 (Atg16L in mammals) to form the Atg12–Atg5–Atg16 complex [16]. The second reaction occurs at LC3, the homologue of yeast Atg8 in mammals, which is required for lysosome formation. Atg4 cleaves LC3 to obtain LC3-I in the diffuse cytoplasmic state, which is subsequently coupled to phosphatidylethanolamine in order to form LC3-II in the membrane-bound state, which is subsequently localised in the autophagosome membrane [17]. The Atg5–Atg12–Atg16L1 complex associates with pre-autophagosome membranes, prolonging their elongation by assisting in the recruitment of LC3. The Atg5–Atg12–Atg16L1 complex separates from the outside membrane as the phagocyte grows and gets closer to closing, whereas LC3-II stays attached to the finished autophagosome. The autophagosome can bind autophagic substrates and/or proteins that mediate cargo-selective autophagy (such as p62) thanks to LC3 and LC3 homologues. In addition to this, mAtg9 is the only multiple transmembrane protein identified among the core Atg proteins, and phagocytosis is assisted by mAtg9 extension. Meanwhile, organelles such as mitochondria, plasma membrane, and Golgi complex donate their cell membranes to the extended autophagosome membranes, facilitating the elongation, closure, and eventual formation of bilayer vesicles. Mature autophagosomes combine with lysosomes to form autophagic lysosomes, which degrade internal substances, recycle amino acids, fatty acids, and nucleotides, and maintain the dynamic balance of biomolecules and energy in the cell (Figure 2).

### 2.2 Autophagy regulation

Autophagy contributes to the cellular response to a wide range of extracellular and intracellular stresses, including nutrient starvation, the insulin/growth factor pathway, hypoxia, and ER stress. Serine/threonine protein kinase (TOR) pathways and cAMP-dependent protein kinase A (PKA) pathways, there are two well accepted pathways that played key roles in these processes.

#### 2.2.1 Mammalian target of rapamycin (mTOR) pathway

In mammals, Ulk1 as a direct homologue of yeast Atg1 is associated with starvation-induced autophagy, and combine additionally with Atg13, FIP200, and Atg101, Atg13 to form stable complexes that do not change with nutritional status [18]. The rapamycin-targeted protein mTOR is a key regulator of autophagy induction. Only TORC1 directly controls autophagy out of the two functionally different protein complexes that Tor produces, Tor complex 1 and 2 (TORC1 and TORC2) [19]. While mTORC1 is repressed during nutritional deficiency, which boosts autophagic activity, it is active under nutrient-rich situations and suppresses autophagy. The phosphorylation state within the Ulk1–Atg13–FIP200 complex varies significantly with nutritional status. Under high nutritional conditions, active mTOR phosphorylates Atg13 and Ulk1, thereby inhibiting Ulk1 kinase activity [20]. Upon starvation, the mTORC1 site on ULK1 is dephosphorylated and ULK1 is separated from mTORC1. At the same time, ULK1 undergoes autophosphorylation, followed by phosphorylation of ATG13 and FIP200, or, alternatively, Ulk1 is phosphorylated by AMPK and consequently activated [21].

#### 2.2.2 Ras/PKA pathway

Apart from TORC1, the Ras/PKA signalling system controls autophagy in organisms ranging from yeast to humans. The regulatory subunit Bcy1 and three catalytic subunits (Tpk1, Tpk2, and Tpk3) that appear to be redundant make up the heterotetramer that is yeast PKA. In yeast cultured in an enriched medium, two redundant small GTPases called Ras1 and Ras2 are activated, stimulating adenylate cyclase to produce cAMP. They then bind to the PKA regulatory subunit, known as Bcy1, and facilitate its dissociation from three PKA catalytic subunits, Tpk1, Tpk2, and Tpk3, which in turn activates PKA.

Elevated cAMP binds to Bcy1 and releases its inhibitory effect on PKA. Constitutive activation of the Ras/PKA pathway inhibits TOR inhibition-induced autophagy in yeast [22]. Ras/PKA is thus an additional negative regulator of autophagy in addition to TORC1 [23]. It has been shown that PKA and Sch9 signalling pathways synergistically regulate the induction of autophagy, and that simultaneous inactivation of PKA and Sch9 induces autophagy independently of the effect of TORC1, and that inactivation of TORC1 can further increase autophagy [24].

## 3 MSCs

First identified in bone marrow by Friedenstein et al. in 1974, MSCs are multipotent cells with self-renewal capacity and mesodermal origin [25]. As a non-haematopoietic adult stem cell, and with the potential for self-renewal and multidirectional differentiation [26], MSCs is widely found in bone marrow, umbilical cord tissue, umbilical cord blood, peripheral blood, adipose, and other tissues. Under specific induction conditions *in vivo* or *in vitro*, MSCs can differentiate into mesodermal mesenchymal tissue cells such as osteoblasts, adipocytes, chondrocyte, etc., and moreover, are also capable of crossing the embryonic boundary and differentiate into ectodermal neurons, neuroglia, and endodermal hepatocytes.

Although derived from a variety of sources, MSCs share some common characteristics. According to the International Society for Cellular Therapy, MSCs express CD105 (SH2), CD73 (SH3), CD44, and CD90, but not CD45, CD34, CD11b, CD19, and HLA-DR, and can be cultured in adherence and differentiate into the different functional cell types mentioned above [27].

The potential of human pluripotent stem cells, such as embryonic stem cells (ESCs) and induced pluripotent stem cells (iPSCs), in the treatment of cellular injury and recalcitrant diseases is immense, but there are some issues that limit the translation of MSCs to clinical applications, mainly including: their inherent tumourigenicity, immunogenicity, and heterogeneity [28]. Compared with ESCs and iPSCs, MSCs do not have such problems. The easy accessibility, multiple differentiation potentials, good proliferation rate, and safety of clinical application of MSCs make them an ideal class of cells for cell therapy, which have been widely used in experimental studies and clinical settings, and in the field of tissue and organ repair, including bone, cardiac, cartilage, central nervous system, and skin [29].

Restoring damaged cells and organs brought on by different illnesses and harmful substances has proven to be a significant medical problem. A growing body of research indicates that MSCs are capable of mediating tissue and organ repair by differentiating into multiple cell lineages in the microenvironment, and thus may be able to treat a wide range of illnesses and injuries, as well as many of the medical issues that people encounter. However, a variety of internal and external variables influence the biological activities of MSCs following their proliferation and transplantation *in vitro* and *in vivo*, and under certain disease conditions, MSCs’ ability of differentiation also becomes compromised. As a key cytoprotective system, autophagy aids in cell adaptation to shifting conditions, shields cells from harm from external sources, and eventually preserves cellular homeostasis, which may regulate MSCs’ survival and differentiation and achieve better therapeutic effects.

## 4 Autophagy regulates MSC differentiation

To date, MSCs have been widely studied and used in regenerative medicine. However, the mechanisms that determine their efficacy in clinical application are poorly understood. The pluripotent differentiation spectrum potential of MSCs is usually defined as the capacity to differentiate into osteoblasts, chondrocytes, and adipocytes *in vitro* [30]. Different local microenvironments (cell morphology, cytoskeletal tension, cell adhesion, and mechanical or structural cellular properties) can regulate stem cell differentiation towards different lineages. Low-density stem cells tend to differentiate towards osteoblasts, whereas high-density cells make cells susceptible to condensation and differentiate towards lipogenic cells. Many lines of evidence suggest that autophagy plays a major regulatory role in MSCs self-renewal and lineage commitment. It has been shown that autophagy acts in BMSCs to help them switch between being osteogenic and adipogenic, with undifferentiated MSCs displaying a large accumulation of undegraded autophagosomes and little autophagic turnover, whereas osteogenic differentiation of MSCs leads to more autophagic turnover [4]. In contrast, the induction of autophagy in BMSCs may also be responsible for the reduction of their S-phase population and trigger their differentiation into neurons [31]. Nonetheless, it has been proposed that the fundamental differences among osteogenic, lipogenic, and chondrogenic differentiation may be caused by the distinct ways, including but not limited to, Wnt/β-catenin, Notch, and Nrf2 signalling, by which autophagy influences the MSCs’ differentiation [32]. As a negative regulator of basal and stress-induced autophagy, the Wnt/β-catenin signalling pathway is activated in MSCs by stimulating osteogenesis and inhibiting adipogenesis [33]. The Notch signalling pathway is involved in regulating the cell differentiation, and Song et al. found that Notch signalling was inhibited in an autophagy-dependent manner during the process of MSCs lipogenic differentiation, and the inhibition of autophagy along with the inhibition of adipogenesis could induce Notch activation [34]. NRF2 is an important transcription factor in the maintenance of cellular redox homeostasis, and binds to Kelch-like ECH-associated protein 1 (Keap1) to undergo proteasomal degradation under basal conditions. P62 in the autophagy machinery is able to bind to Keap1 to promote the antioxidant function of NRF2. Tao et al. found that under oxidative stress conditions, inhibition of autophagic activity led to excessive accumulation of P62, resulting in activation of the NRF2 pathway, whereas osteoblastic differentiation of MSCs was inhibited at this time [35]. Therefore, how autophagy specifically intervenes in different differentiation directions of MSCs should be further studied, and the exact signalling pathways need in-depth exploration.

Based on the regulation of autophagy on MSCs, promising opportunities exist for enhancing the effectiveness of MSC transplantation in cell-based medical therapy and regenerative medicine. Inspired advancements *in vitro* and *in vivo* have been reported in studies of MSC-based cell therapy for bone abnormalities, including the placement of scaffolds seeded with MSCs into the locations of bone defects, and treat alveolar cleft deformities to rebuild jaw defects [36,37]. Because of the difficulties of grafts for osteochondral transplantation and the fibrocartilage regeneration, cartilage repair remains one of the major challenges in medicine. Therefore, the promotion of the differentiation of MSCs into chondrocytes for repairing cartilage defects holds better promises than traditional repair techniques [30]. Although there are no clinically useful ways to heal nervous system diseases, dental pulp- and umbilical cord blood-derived MSCs exhibit excellent neuronal differentiation properties and could be induced into neuron-like cells *in vitro*, MSCs-based neurorestorative properties have shed light on the treatment of diseases and damages of nervous system [38,39]. Here, we provided a specific description of regulation of MSCs from the perspective of autophagy to promote the MSCs’ differentiation, thus offering wider application of MSCs in regenerative medicine.

**Figure 3:**
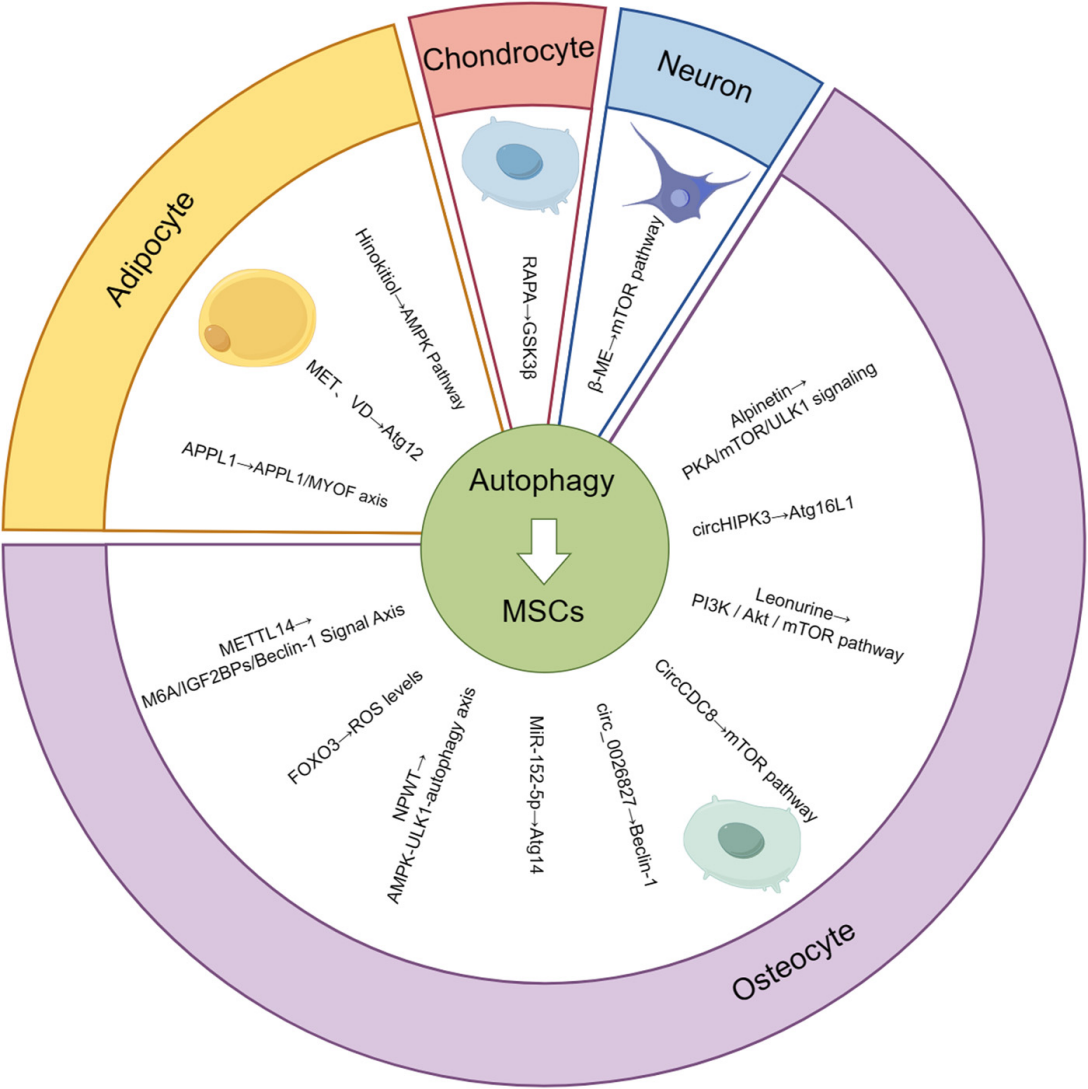
Complex associations exist between autophagy and MSCs differentiation induced by various extracellular or intracellular signals. This figure was generated by Figdraw.

Autophagy is a complex process, and its impact on MSCs differentiation has been extensively studied and progressed in recent decades, but its influencing factors and related mechanisms are not yet fully understood, as detailed below (Table 1, Figure 3).

**Table 1:** Factors and mechanisms affecting MSCs differentiation via the autophagy pathway

Factor	MSCs type	Environmental conditions	Autophagy change	Differentiation	Mechanisms	References
Negative pressure wound therapy	Rat MSCs	—	Activation	Osteogenic↑	AMPK-ULK1-autophagy axis↑	[40]
Rapamycin	Mice BMSCs	Osteoporotic mice	Activation	Osteogenic↑	—	[41]
FOXO3	Human BMSCs	—	Activation	Osteogenic↑	Activation of autophagy downregulates ROS levels	[42]
Methyltransferase-like 14	Mice BMSCs	Osteoporotic mice	Activation	Osteogenic↑	M6A/IGF2BPs/Beclin-1 signal axis↑	[44]
Galectin-3-tripartite motif protein 16 (TRIM16)	Human BMSCs	Osteoporotic mice	Activation	Osteogenic↑	ULK1-dependent manner	[46]
MiR-152-5p↓	Rat mandible MSCs	Osteoporotic rats	Activation	Osteogenic↑	Negative regulation Atg14	[47]
MiR-140-3p↓	Rat BMSCs	—	Activation	Osteogenic↑	Targeting spred2	[48]
MiR-3-223p↓	Human BMSCs	—	Activation	Osteogenic↑	Targeting FOXO3	[49]
circHIPK3↓	Human BMSCs	—	Activation	Osteogenic↑	Stable binding of HUR-ATG16L1↑	[50]
circ_0026827↑	DPSCs	—	Activation	Osteogenic↑	miR-188-3p↑, Beclin-1↑	[51]
CircCDC8↑	PDLSCs	Anoxic conditions	Activation	Osteogenic↓	mTOR pathway	[52]
HDAC9↓	Mice BMSCs	Aging osteoporosis	Activation	Osteogenic↑	—	[53]
Cav 1.3↑	Rat BMSCs	Osteoporotic rats	Inhibition	Osteogenic↓	Negative regulation spred2	[54]
Leonurine	Rat BMSCs	Osteoporotic rats	Activation	Osteogenic↑	PI3K/Akt/mTOR pathway	[55]
Alpinetin	Mice BMSCs	Mouse model of osteoporosis	Activation	Osteogenic↑	PKA/mTOR/ULK1 signalling	[56]
TPPU	DPSCs	Inflammatory conditions	Inhibition	Osteogenic↑	Increased EETs levels	[57]
γ-Aminobutyric acid receptor-associated protein	Rabbit BMSCs	Inflammatory conditions	Activation	Osteogenic↑	ROS↓	[58]
Scara3	Mice BMSCs	Osteoporotic mice	Activation	Adipogenic↓osteogenic↑	FOXO1↑	[59]
Adaptor protein containing pH domain, PTB domain, and leucine zipper motif 1 (APPL1)	Human BMSCs	Osteoporotic mice	Inhibition	Adipogenic↑	APPL1/MYOF axis	[61]
Hinokitiol	MSCs	—	Inhibition	Adipogenic↓	AMPK pathway	[62]
Fluoxetine	Human adipose-derived MSCs	—	Activation	Adipogenic↓	—	[63]
Metformin and Vitamin D	Human adipose-derived MSCs	—	Inhibition	Adipogenic↓	Atg12↑	[64]
Articular chondrocyte-derived extracellular vesicles	Umbilical cord MSCs	—	Activation	Cartilage↑	—	[65]
Rapamycin	Human synovium-derived MSCs	Inflammatory conditions	Activation	Cartilage↑	GSK3β	[66]
Low-intensity pulsed ultrasound	Rat BMSCs	—	Inhibition	Cartilage↑	—	[67]
5-Azacytidine	Human tonsil-derived MSCs	—	Activation	Myogenic↑	—	[68]
DAPT, 5-azacytidine	Human placenta-derived MSCs	—	Activation	Neural↑	—	[69]
β-Mercaptoethanol	Rat BMSCs	—	Activation	Neural↑	mTOR pathway	[70]

### 4.1 Osteogenic differentiation

Autophagy plays a crucial role in mediating the differentiation of MSCs, thereby facilitating bone homeostasis. Zhang et al. [40] cited negative pressure wound therapy, in which MSCs in negative pressure hydrogel in a rat model of critical-size cranial defects induced osteoblastic differentiation of rat MSCs by activation of autophagy through the phosphorylation of AMPK in ULK1 and it was found that this AMPK activation-driven autophagy is required in the NP-mediated osteogenic differentiation. Low bone mineral density, excessive microarchitecture, and a higher risk of fragility fractures are the hallmarks of osteoporosis. Impaired autophagy is frequently linked to the onset of osteoporosis. Qi et al. [41] found that reduced autophagy was closely associated with osteoporotic BMSCs exhibiting impaired osteogenic differentiation by establishing a model of osteoporotic mice, as well as affecting their inhibitory immunotherapeutic properties, and treatment with rapamycin reactivated autophagy to alleviate the osteoporotic phenotype in mice, suggesting that activation of autophagy contributes to osteogenic differentiation of BMSCs. Under conditions of oxidative stress, H_2_O_2_-treated MSCs activate Forkhead box O3 (FOXO3), which then induces autophagy in response to elevated levels of reactive oxygen species (ROS), thereby preventing oxidative damage. Consistently, inhibition of autophagy impairs ROS clearance and the osteogenic capacity of MSCs [42].

In the case of oxidative damage, these cytoprotective effects of autophagy on MSCs seem to depend on the severity and duration of the stress. In the early stages of H_2_O_2_-induced MSCs injury, autophagic flux is considered as a self-defence process and this protective effect disappears after prolonged oxidative exposure [43]. Methyltransferase-like 14 is essential for autophagy activation and suppresses osteoporosis. He et al. discovered that methyltransferase-like 14 overexpression markedly increases bone production and slows osteoporosis progression in an established rat model with ovariectomized hips [44]. For the mechanisms, with the increasing Beclin-1 m6A modification level, the autophagy signalling pathway was triggered and significantly the osteogenic differentiation of BMSCs was enhanced. Galectin-3 expression was found to be upregulated like other osteoblast markers in studies of long-term osteogenic differentiation. Chauhan and colleagues found that Galectin-3 can interact with TRIM16, and TRIM16 further binds to ATG16L1, and to ULK1+ Beclin1 binding induces lysosomal and phagosomal damage during autophagy [45]. Chen et al. further found that the interaction between Galectin-3 and TRIM16 through autophagy could promote osteogenic differentiation of bone marrow MSCs [46].

A type of small, naturally occurring non-coding RNA known as microRNAs controls the expression of genes post-transcriptionally. Recent research has shown that microRNAs are essential for controlling autophagy in relation to homeostasis and bone formation. In a rat model of mandibular osteoporosis, the researchers found that down-regulation of miR-152-5p activated autophagy to stimulate the osteogenic differentiation of OVX mandibular MSCs by targeting ATG14 [47]. By directly integrating with the 3′-UTR of Spred2 mRNA, miR-140-3p was able to target Spred2, as reported by Liu et al. [48]. This resulted in an inhibition of miR-140-3p expression, which in turn promoted osteogenic differentiation by activating the autophagy pathway, suggesting that miR-140-3p negatively regulated the osteogenic differentiation of BMSCs. MiR-223-3p mimics, inhibitors, FOXO3 overexpression plasmids, or lentiviral carriers of siFOXO3 were transfected into BMSCs to detect the expression of autophagy- and osteogenesis-related genes, and found that up-regulation of miR-223-3p inhibited the expression of BMSCs autophagy-related gene expression and down-regulated the protein and mRNA of osteogenic differentiation-related genes, whereas overexpression of FOXO3 reversed this effect, suggesting that miR-3-223p-targeted FOXO3 can promote osteogenic differentiation of BMSCs by enhancing autophagy [49].

The involvement of circular RNAs in the osteogenic differentiation of MSCs has also been widely reported. circHIPK3 has recently been used to explore the link between autophagy and the osteogenic differentiation of MSCs. When circHIPK3 was knocked down by transfection small interfering RNA, the HUR and ATG16L1 binding site was released, leading to upregulation of ATG16Ll and autophagy activation to promote osteogenesis in MSCs [50]. The function of circ_0026827 in dental pulp stem cells (DPSCs) osteoblast development was examined by Fang et al. With DPSCs osteogenic differentiation, circ_0026827 expression increased. Luciferase reporter gene assays verified that miR-188-3p was its target, and RUNT-associated transcription factor 1, by targeting Beclin-1-mediated autophagy, promoted DPSCs osteoblast differentiation [51]. circCDK8, a circRNA located at the gene for cell cycle protein-dependent kinase 8, is able to activate autophagy in periodontal ligament stem cells (PDLSCs) via the mTOR signalling pathway under hypoxic conditions, but differently, the activation of autophagy inhibits osteogenic differentiation in PDLSCs [52].

Bone loss during aging is associated with an imbalance in the lineage differentiation of bone BMSCs. During aging, MSCs are subject to epigenetic and transcriptional changes, and histone deacetylases (HDACs) are important epigenetic regulators. According to Zhang et al., HDAC9 is crucial for preserving the equilibrium between adipogenesis and osteogenesis in BMSCs during age-related bone loss, via regulation of autophagy, HDAC9 impaired differentiation of aged BMSCs, whereas inhibition of HDAC9 ameliorated bone loss in aged mice [53]. In senile osteoporosis, cellular senescence may be a further factor that affects the differentiation of osteoblasts. Fan et al. first found that Cav 1.3 negatively regulates the effects of Spred2 in inhibiting cell cycle arrest and cellular senescence in BMSCs from OS rats by activating autophagy and ultimately impairs BMSCs activity and osteogenic differentiation in OS rats [54].

Traditional Chinese herbs and their extracts for the prevention and treatment of osteoporosis are of increasing interest, have fewer side effects than synthetic drugs and show greater sustainability in the long term. Zhao et al. reported a Chinese herbal extract, leonurine, which can activate autophagy by inhibiting the PI3K/Akt/mTOR pathway that promotes BMSCs osteogenic differentiation without significant cytotoxicity [55]. By boosting PKA/mTOR/ULK1 autophagy signalling, the phytochemical alpinetin enhanced osteogenic differentiation of BMSC and dramatically reduced dexamethasone-induced bone loss in a rat model of osteoporosis [56].

Under inflammatory conditions, autophagy has a dual role. In the early stages of inflammation, autophagy protects cells from damage and hinders the induction of apoptosis, whereas in the later stages, excessive accumulation of autophagosomes may have the opposite effect. Dysfunction of autophagy impairs the ability of MSCs to differentiate and increases inflammation-induced bone loss. Epoxyeicosatrienoic acids (EETs) have been linked to autophagy and have been demonstrated to have strong anti-inflammatory properties. Dang et al. promoted osteogenic differentiation of DPSCs by increasing the level of EETs on this basis. According to research by Dang et al., inflammatory circumstances brought on by lipopolysaccharide elevated inflammatory cytokines, prevented DPSCs from differentiating into osteoblasts, and caused DPSCs to undergo excessive autophagy. When EET levels were increased with 1-(4-trifluoromethoxyphenyl)-3-(1-propionylpiperidin-4-yl)urea (TPPU), a soluble epoxide hydrolase inhibitor to increase the levels of EETs, these negative results were reversed. Experiments showed that the presence of TPPU attenuated LPS-induced excessive autophagy and restored the osteogenic differentiation potential of DPSCs in an inflammatory microenvironment [57]. Guo and Wu mimicked interleukin-1β (IL-1β)-induced inflammatory conditions, and autophagy of pre-γ-aminobutyric acid receptor-associated protein increased BMSCs viability and proliferation by limiting intracellular ROS generation through up-regulation of autophagy restriction and promoted osteogenic differentiation of BMSCs *in vitro* [58].

### 4.2 Adipogenic differentiation

Scavenger receptor class A, member 3 (Scara3) has recently been used as an entry point to study the regulation of transitions between adipocyte and osteoblast differentiation. Scara3 is positively correlated with osteogenesis-related genes, and was found to be a key regulator involved in BMSCs adipogenesis and osteogenesis, and it activates autophagy by positively correlating with the expression of FOXO1 thereby affecting BMSCs differentiation. In an *in vivo* study, overexpression of Scara3 attenuated bone loss in osteoporotic mice [59]. The autophagy-specific receptor optineurin is also shown to be closely related to the selection of osteogenic or lipogenic differentiation in senescent MSCs. Optineurin is able to reduce the probability of osteoporosis during aging by removing fatty acid binding protein 3, muscle, and heart, which has the ability to promote adipogenesis and inhibit the osteogenic function of MSCs [5]. In addition, the lysosomal subpopulation of the chaperone-mediated autophagy lysosome promotes osteogenesis by selectively removing osteogenic inhibitory factors controlled by lysosomal Van-Gogh-like 2 (Vangl2), which binds to and promotes the degradation of lysosome-associated membrane protein 2A (LAMP-2A). It is also found that the ratio of Vangl2/LAMP-2A is elevated during osteogenic differentiation and decreased during lipogenic differentiation, thus the Vangl2–LAMP-2A axis can be used to fine-tune lysosomal activity and influence MSCs lineage selection [60].

Adaptor protein containing pH domain, PTB domain, and leucine zipper motif 1 (APPL1) is an articulatory protein of the lipocalin receptor, which has recently been shown to be associated with the lipid differentiation of MSCs in osteoporosis. In Zhang et al.’s study [61], APPL1 binds to and inhibits the ubiquitin-mediated degradation of the downstream target protein, MYOF, and stabilises lysosomal function during MSCs lipid differentiation. When APPL1 is deficient, the normal degradation of lysosomal autophagy is impaired, leading to reduced autophagic flux and promotion of MSCs lipid differentiation. Lee et al. [62] reported that when MSCs were pretreated with platanol, a natural mono ketone compound, platanol, that in platanol-treated MSCs, the level of LC3-II was reduced, while the level of p62 was increased, and the phosphorylation of AMPK was increased in a concentration-dependent manner, decreasing their differentiation to mature adipocytes. Autophagy activator both rapamycin treatment as well as inhibition of AMPK phosphorylation altered this outcome. This suggests that flatulence-mediated AMPK activation and inhibition of autophagic flux inhibit lipid accumulation and differentiation of MSCs into adipocytes and also suggests that autophagic flux modulators and AMPK signalling including flatulence can be used to regulate differentiation of MSCs [62].

Autophagy has been shown to regulate fat mass and differentiation, and inhibition of autophagy in preadipocytes decreases triglyceride accumulation and expression of transcription factors involved in adipocyte differentiation. Sun et al. found that fluoxetine was able to increase the expression of autophagy-related genes (e.g., SQSTM1 and LC3B), which reduced adipose accumulation by activating autophagy to inhibit adipose-derived stem cells proliferation and differentiation [63]. When adipose-derived stem cells were cultured in the presence of vitamin D or metformin or both, the number of mature adipocytes was reduced which may be related to the inhibition of autophagosome formation by high expression of Atg12 protein [64].

### 4.3 Chondrogenic differentiation

Pluripotent MSCs have become a promising tool in stem cell-based techniques for cartilage regeneration; therefore, it is highly valuable to do research on how to promote MSCs’ chondrogenic differentiation through the autophagy route. Extracellular vesicles (EVs) have been reported to regulate the phenotypic expression of stem cells. In a rabbit cartilage defect model, MSC-EVs induced the chondrogenic differentiation of human umbilical cord MSCs through the activation of autophagy in it to promote the repair of cartilage defects [65]. MSCs show better chondrogenesis and are a promising cell type for tissue engineering of cartilage. In addition to decreasing the LC3-II/LC3-I ratio and autophagosome formation, IL-1β increases the expression of mTOR. Rapamycin promoted autophagy and improved MSCs chondrogenic differentiation [66]. However, the role of autophagy in MSCs chondrogenic differentiation was not consistent. In bone marrow MSCs, Wang et al. found that low-intensity pulsed ultrasound promoted MSCs cartilage formation by inhibiting autophagy [67].

### 4.4 Other differentiation directions

Skeletal muscle development, regeneration, and homeostasis have all been shown to be significantly regulated by autophagy. Uncontrolled autophagy has also been linked to sarcopenia brought on by aging and muscle diseases. Tonsillar MSCs undergo myogenic differentiation through autophagic activity. The autophagy inhibitor bafilomycin A1 reduces the expression of myogenic markers, while the autophagy inducer 5-azacytidine encourages MSCs development into myoblasts and skeletal muscle cells [68]. Sotthibundhu et al. [69] studied human placenta-derived MSCs, where Notch signalling plays a key role in guiding the type of stem cell differentiation. Using the Notch signalling pathway inhibitor DAPT in conjunction with the autophagy inducer 5-azacytidine, they up-regulated the expression of neuronal genes as well as the autophagy genes LC3I/II and Beclin in human placenta-derived MSCs, whereas inhibition of autophagy prevented neural differentiation. Li and their team carried out a study on autophagy and neuronal differentiation of MSCs. Their observations indicated that autophagy was activated during the neuronal differentiation of MSCs, and that appropriate mTOR activity could enhance the efficiency of neuronal differentiation [70].

Current research suggests that it makes sense to treat diseases by regulating autophagy to increase the efficiency of MSCs differentiation, but the exact mechanisms underlying the relationship between autophagy and MSCs differentiation are complex and require further investigation. Autophagy has recently garnered recognition as a vital cellular function for safeguarding stem cells against external harm. Interestingly, even though a lot of regular cell types need some level of autophagic regulation, any circumstance that the cell cannot manage may set off a particular dying mechanism: demise of autophagic cells [71]. This double-edged nature also adds more demanding conditions for research. Meanwhile, the current studies on autophagy regulation of MSCs differentiation exist more at the cellular level, with few *in vivo* studies. The *in vivo* environment is a dynamic one in which many pathways and cells are in constant exchange and communication, and the complexity of such potential interactions is difficult to predict from *in vitro* studies. In this review, it can be seen that many studies have been devoted to seeking and validating intra- and extracellular stimuli that can modulate autophagy to control the fate of MSCs, but great efforts are still needed to precisely regulate autophagy in a way that contributes to the treatment of MSCs.

## 5 Summary and outlook

Among the factors that determine the fate of MSCs, autophagy has received much attention, and its activation is usually stimulated by different conditions, including cell starvation, inflammation, oxidative stress, and some others [72]. Autophagy is a process by which cells degrade and recycle themselves, and through which MSCs may mobilise autophagic degradation to induce protein production and recycling of components of other key energy factors [73]. Moreover, autophagy is mobilised at an early stage of MSCs differentiation [4], confirming that the involvement of autophagy has a non-negligible role in this process. Thus, altering the autophagic state at early stages of differentiation alters the long-term differentiation efficiency of MSCs. Regulation of autophagy in MSCs represents a potential strategy to influence the characteristics of MSCs and enhance their regenerative potential in terms of differentiation properties and implantation capacity. Overall, the prevailing view supports the hypothesis that autophagy contributes to maintaining the integrity of MSCs by maintaining their self-renewal and differentiation potential.

Furthermore, autophagy is a double-edged sword, with its impact varying according to the characteristics, severity, and duration of a given stress [6]. In general, autophagy plays a protective role in cells, but disruption of the autophagic machinery or excessive autophagic flux can also have the opposite effect. Initial studies suggested that autophagy encourages MSCs senescence, and elevated glucose levels have been found to hasten senescence by increasing MSCs autophagy and causing ROS production [74]. Moreover, it is believed that autophagic activity is required to keep MSCs in the senescent state after they enter it. When human MSCs were co-cultured with primary chondrocytes, autophagy is responsible for the disappearance of MSCs to contribute to their trophic effects [75].

Collectively, it was found that autophagy plays an important role in the differentiation of MSCs both at the cellular and molecular levels. However, further in-depth studies are needed on the impact of autophagy on MSCs differentiation and the underlying molecular mechanisms. In this way, research on autophagy in directed differentiation of MSCs will become a breakthrough point in stem cell engineering, laying a foundation for the clinical application of MSCs.
